# In-Field Tobacco Leaf Maturity Detection with an Enhanced MobileNetV1: Incorporating a Feature Pyramid Network and Attention Mechanism

**DOI:** 10.3390/s23135964

**Published:** 2023-06-27

**Authors:** Yi Zhang, Yushuang Zhu, Xiongwei Liu, Yingjian Lu, Chan Liu, Xixin Zhou, Wei Fan

**Affiliations:** 1 College of Bioscience and Biotechnology, Hunan Agricultural University, Changsha 410128, China; yzhang2020@hunau.edu.cn (Y.Z.);; 2 College of Food Science and Technology, Hunan Agricultural University, Changsha 410128, China

**Keywords:** leaf maturity, in situ recognition, lightweight CNN, Feature Pyramid Network (FPN), attention mechanism, deep learning, precision agriculture

## Abstract

The maturity of tobacco leaves plays a decisive role in tobacco production, affecting the quality of the leaves and production control. Traditional recognition of tobacco leaf maturity primarily relies on manual observation and judgment, which is not only inefficient but also susceptible to subjective interference. Particularly in complex field environments, there is limited research on in situ field maturity recognition of tobacco leaves, making maturity recognition a significant challenge. In response to this problem, this study proposed a MobileNetV1 model combined with a Feature Pyramid Network (FPN) and attention mechanism for in situ field maturity recognition of tobacco leaves. By introducing the FPN structure, the model fully exploits multi-scale features and, in combination with Spatial Attention and SE attention mechanisms, further enhances the expression ability of feature map channel features. The experimental results show that this model, with a size of 13.7 M and FPS of 128.12, performed outstandingly well on the task of field maturity recognition of tobacco leaves, achieving an accuracy of 96.3%, superior to classical models such as VGG16, VGG19, ResNet50, and EfficientNetB0, while maintaining excellent computational efficiency and small memory footprint. Experiments were conducted involving noise perturbations, changes in environmental brightness, and occlusions to validate the model’s robustness in dealing with the complex environments that may be encountered in actual applications. Finally, the Score-CAM algorithm was used for result visualization. Heatmaps showed that the vein and color variations of the leaves provide key feature information for maturity recognition. This indirectly validates the importance of leaf texture and color features in maturity recognition and, to some extent, enhances the credibility of the model. The model proposed in this study maintains high performance while having low storage requirements and computational complexity, making it significant for in situ field maturity recognition of tobacco leaves.

## 1. Introduction

The maturity of tobacco leaves is pivotal in determining the quality and characteristics of cigarette products [1], thereby becoming a key factor that affects the quality of the leaves. Not only does it correlate with the chemical composition, aroma, and flavor of tobacco leaves [2,3], but it also considerably influences the sustainability of the tobacco industry and the economic welfare of tobacco farmers [4,5]. Maturity serves as a critical indicator determining the harvesting period for tobacco leaves [6]. However, prevailing manual classification methods are inefficient, costly, and susceptible to bias. Overripe or underripe tobacco leaves may lead to decreased yield, reduced quality, and increased difficulty in curing [7], thereby impacting the earnings of tobacco farmers and the overall competitiveness of the tobacco industry. Therefore, accurately determining the optimal harvest time for tobacco leaves and establishing an objective tobacco leaf maturity evaluation system is vital for elevating the quality of tobacco leaf production, aiding tobacco farmers in maximizing profits, and maintaining the stable development of the tobacco industry while securing high-quality tobacco leaves [8].

In recent years, researchers have ventured into exploring various technologies to measure the maturity of tobacco leaves. Yu et al. [9] employed electrochemical fingerprint technology to classify tobacco types and monitor the growth status of tobacco leaves. Conversely, Chen et al. [10] amalgamated near-infrared (NIR) spectroscopy with convolutional neural network (CNN) deep learning to achieve maturity recognition of the upper, middle, and lower parts of the tobacco leaves, reaching accuracies of 96.18%, 95.2%, and 97.31%, respectively. Concurrently, Lu et al. [11] utilized hyperspectral imaging technology combined with the SNV-SPA-PLS-DA model, successfully increasing the accuracy of the tobacco leaf maturity classification validation set and prediction to 99.32% and 98.46%, respectively. These findings underscore the efficacy of utilizing visible light/NIR hyperspectral imaging technology to detect tobacco leaf maturity. However, these methods recognize tobacco leaf maturity after harvesting, leading to the destruction of the leaves. Although spectral technology offers non-destructive identification of tobacco leaf maturity, spectrometers are expensive, lack portability, and are susceptible to environmental interference. With the swift evolution of machine vision and smart sensing technology, these methodologies showcase substantial potential in agricultural arenas such as plant disease detection, fruit maturity evaluation, and yield prediction, thus offering more convenient and efficient solutions for agricultural operations [12,13]. Mallikarjuna et al. [14] proposed a tobacco leaf selective harvesting method based on texture features, validating the effectiveness of texture analysis in distinguishing mature from immature leaves. Moreover, color features play an integral role in tobacco leaf maturity recognition [15]. Drawing on these studies, Mallikarjuna et al. [16] proposed a maturity evaluation method combining filters and color models, effectively evaluating tobacco leaf maturity, thereby assisting in the development of efficient automatic harvesting systems. Nonetheless, research on tobacco leaf maturity recognition in the field under complex backgrounds and variable weather conditions is still limited. Traditional feature extraction methods may have limited accuracy and generalization capabilities under these conditions [17,18,19,20,21]. Compared with traditional machine learning methods, deep learning methods, particularly convolutional neural networks (CNNs), have marked advantages in image classification [22,23] and object detection tasks, being able to automatically learn and extract image features [24]. By leveraging the ubiquity and convenience of mobile devices in tandem with deep learning technology, Chen et al. [25] established a practical solution for evaluating tobacco leaf maturity. Although Li et al. [26] proposed an improved lightweight deep learning network architecture for recognizing tobacco leaf maturity to surmount the limitations of traditional methods, their research was still conducted post-harvest and does not fully replicate the in situ working conditions of field tobacco leaves. Therefore, the development of a high-accuracy tobacco leaf maturity recognition method suitable for complex environments still holds significant value.

High-definition lenses and optical sensors are capable of capturing the rich texture and spectral features of crops [27]. Hence, in response to the limited research on in situ tobacco leaf maturity recognition in the field, this study leveraged a mobile phone as an information collection tool, proposing a novel method that integrates machine vision with the MobileNetV1 deep convolutional network for in situ tobacco leaf maturity recognition research in the field. This study maintained the authenticity of field operation conditions during the process of tobacco leaf image collection. To accommodate the size differences between tobacco leaves, we introduced the Feature Pyramid Network (FPN) structure to capture and merge features of different scales, thereby enhancing feature expression capabilities. By integrating the attention mechanism, we focus on key information, maintaining high recognition performance in complex backgrounds and weather environments. Further, an improved MobileNetV1 model was used for training and validation to ensure its accuracy and robustness under different environmental conditions. The proposed method’s advantages and limitations were evaluated by comparing it with other technologies. This study aimed to overcome the limitations of sensory evaluation of tobacco leaf maturity levels, provide accurate, reliable, and scientific aids for tobacco leaf harvesting, and establish a maturity discrimination model.

## 2. Material and Methods

### 2.1. In-Situ Tobacco Leaf Sample Collection in the Field

**Figure 1:**
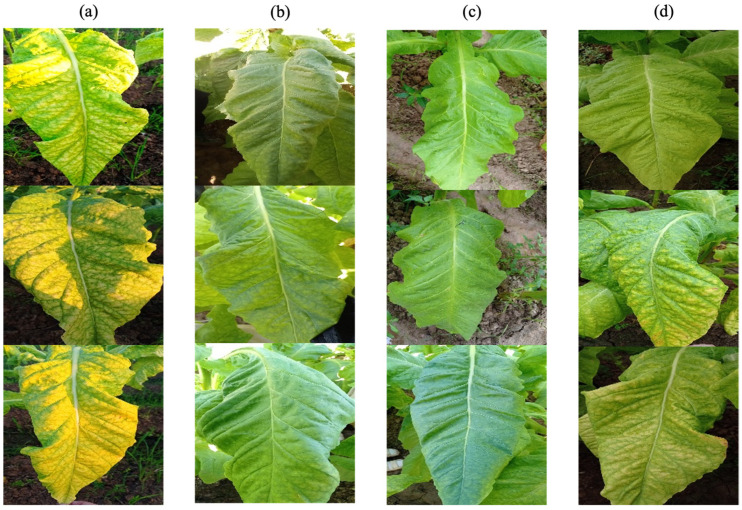
Example images captured under various weather conditions: (**a**) direct sunlight on a sunny day, (**b**) backlighting on a sunny day, (**c**) on an overcast day, and (**d**) on a rainy day.

This study was conducted in Mashui Town, Leiyang City, Hunan Province, China, in 2022, using the tobacco variety Yun87 as the subject. The experiment was carried out from the 100th to the 140th day after the tobacco seedlings were transplanted. Visible light images of tobacco leaves were captured in natural conditions using a Redmi 10X smartphone. This device is equipped with four rear cameras, namely a 48-megapixel main camera, an 8-megapixel ultra-wide-angle lens, and two 2-megapixel lenses for macro photography and portrait depth, respectively. To ensure optimal image capture, the smartphone was set to automatic exposure and autofocus. During the image collection process, no particular effort was made to differentiate between shooting times and weather conditions in order to accurately reflect the field working environment. Whole leaf images were captured directly under natural light at the tobacco cultivation site, collecting image data under various weather backgrounds, including overcast, sunny, and rainy days, as shown in Figure 1. All collected data were categorized according to the maturity assessment criteria found in the literature [28,29] and the experience of local professionals who have long been engaged in tobacco leaf production. In total, 2160 fresh tobacco data samples were collected, divided into nine categories: 251 samples of upper leaves that were under-mature, 255 mature, 219 over-mature; 248 of middle leaves that were under-mature, 279 mature, 194 over-mature; and 215 of lower leaves that were under-mature, 298 mature, 200 over-mature.

### 2.2. Training, Validation, and Test Set Division

To evaluate the performance of the enhanced MobileNetV1 model in recognizing tobacco leaf maturity, the dataset was divided into training, validation, and test sets at a ratio of 7:2:1. The training set was used for model training, the validation set for adjusting the model’s hyperparameters and preliminary performance evaluation, and the test set for assessing the model’s final performance. Stratified sampling was used during the division process to ensure a uniform distribution of data from each category across the datasets.

### 2.3. Data Preprocessing and Augmentation

A range of data augmentation techniques were utilized to boost the performance of the deep learning convolutional neural network in fresh tobacco identification. This encompassed rotation, mirroring, brightness adjustment, and noise perturbation. These manipulations serve to not only effectively deter the model’s overfitting but also enhance its robustness and generalizability. Moreover, data augmentation can help overcome classification issues arising from varying shooting angles. After data augmentation, the 1511 fresh tobacco instances in the training set expanded to 9066, thus amplifying the diversity and volume of the training data.

To conform to the input prerequisites of the MobileNetV1 model, the collected fresh tobacco images underwent geometric transformation using a linear interpolation algorithm and were standardized to 224 × 224-pixel RGB images. This procedure aims to preserve image information while curtailing computational complexity and memory consumption. It also ensures that other pre-trained models used in subsequent experiments can accurately receive and process input data, thus enhancing recognition accuracy.

### 2.4. Tobacco Leaf Maturity Recognition Model Based on Improved MobileNetV1

#### 2.4.1. Structure of the MobileNetV1 Model

MobileNetV1, proposed by Howard et al. in 2017, is an efficient and lightweight CNN architecture designed to provide high-performance visual applications for mobile and embedded devices [30]. Compared to traditional CNN models such as VGGNet and ResNet, MobileNetV1 optimizes computational complexity and memory requirements while ensuring high accuracy [31]. This makes MobileNetV1 better adapted and able to perform in resource-constrained environments like mobile devices and edge computing platforms.

**Figure 2:**
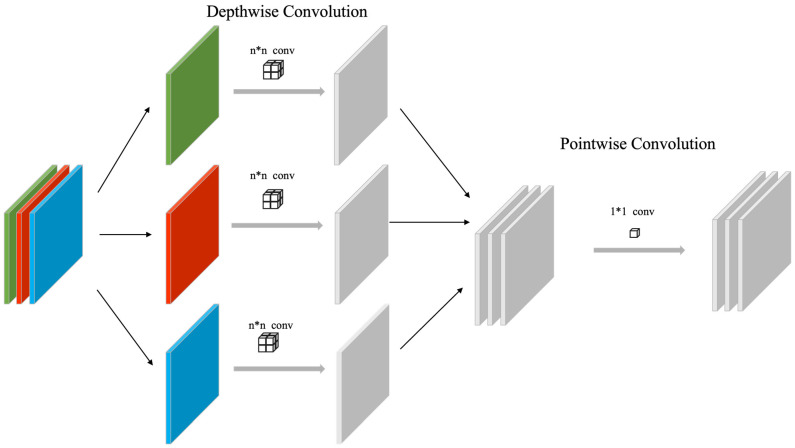
The operation of depthwise separable convolution.

A key innovation of MobileNetV1 is the use of depthwise separable convolution as its main building block. As shown in Figure 2, depthwise separable convolution decomposes the traditional convolution operation into two independent steps: one is depthwise convolution, which deals with local information of input channels, and the other is pointwise convolution, which integrates information from different channels. In this way, depthwise separable convolution significantly reduces computational complexity and the number of model parameters, making MobileNetV1 cost-effective in computation-limited scenarios.

The MobileNetV1 network structure includes a standard convolution layer (Conv Std), thirteen depthwise convolution layers (Conv dw) and thirteen pointwise convolution layers (Conv pw), an average pooling layer (Avg Pool), and a fully connected layer (FC). After each convolution layer, a batch normalization (BN) layer and a rectified linear unit (ReLU) are configured to enhance the model’s expressiveness and convergence speed. To further optimize the number of model parameters, MobileNetV1 uses a global average pooling layer (GAP) to replace the traditional fully connected layer. In this study, the use of MobileNetV1 as the base model can enhance the real-time functioning and feasibility of fresh tobacco recognition, which is more in line with the actual needs of field operations.

#### 2.4.2. Incorporating FPN for Feature Pyramid Construction

The FPN [32] is an effective method for multiscale object detection, capable of extracting and fusing information from feature maps of different scales to capture the information of objects at various scales and complexities. This allows the model to utilize both high-level abstract information (such as the overall shape of the object) and low-level detail information (such as texture and edges) for classification. This is extremely beneficial for dealing with the complex background of in situ tobacco leaf maturity recognition in the field. FPN realizes cross-scale feature fusion through a top-down path and lateral connections, thus enhancing the model’s generalization ability.

In FPN, due to the larger size of low-level feature maps and the smaller size of high-level feature maps, upsampling operations are required to unify the resolution of all feature maps to the same size. Upsampling is a common operation that can be achieved through methods such as bilinear interpolation or transposed convolution. The upsampling operation is carried out in the downsampling branch of the feature pyramid. Specifically, starting from the feature map at a higher level, each level of the feature map increases its resolution through the upsampling operation. During the upsampling process, each feature map is fused with the corresponding upsampled lower resolution feature map to retain high-resolution and rich semantic information. The fused feature map continues to upsample until it reaches the desired resolution.

**Figure 3:**
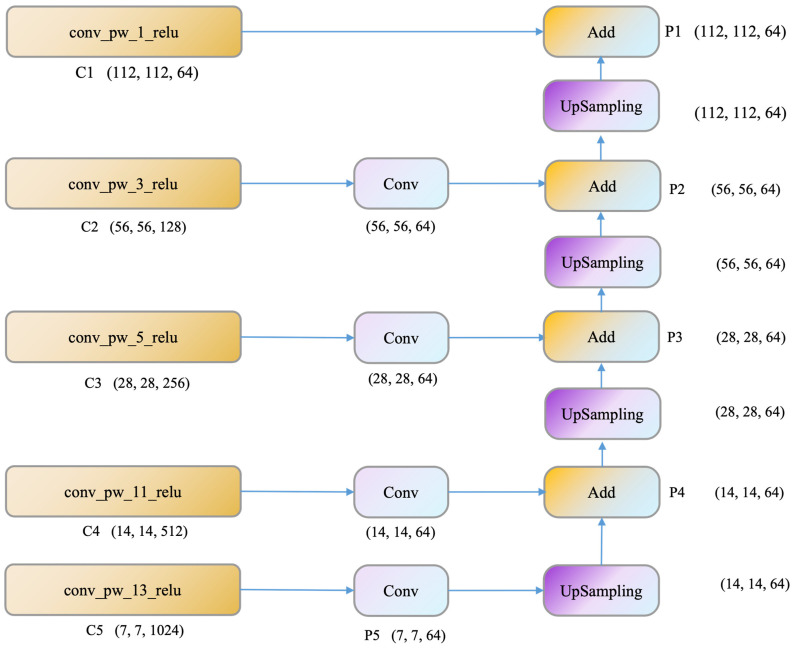
Partial structure of MobileNetV1 integrated with Feature Pyramid Network (FPN).

In the context of this study, MobileNetV1 was used as the base network. As shown in Figure 3, feature maps are extracted from the 1st, 3rd, 5th, 11th, and 13th convolutional layers (namely conv_pw_1_relu, conv_pw_3_relu, conv_pw_5_relu, conv_pw_11_relu, conv_pw_13_relu), denoted as C1, C2, C3, C4, and C5. The extracted feature maps are each subjected to 1 × 1 convolution operation, which effectively reduces dimensions and normalizes the number of channels between different levels, matching them with the number of channels of the first convolutional layer (i.e., 64). Subsequently, the feature maps are successively upsampled and fused from top to bottom to form the FPN structure, creating feature pyramids P5, P4, P3, P2, and P1. This approach more effectively uses the inherent feature hierarchy of convolutional neural networks and avoids the common over-reliance on high-level features when using MobileNet, ensuring a more comprehensive and efficient use of input image information by the model.

Due to inconsistent capturing poses, the collected tobacco leaf images exhibit variations in size and angles. The FPN addresses this issue by processing the images in a multi-scale manner, enabling simultaneous detection of objects at different scales. This capability is particularly useful for on-field in situ identification of tobacco leaf maturity against complex backgrounds. Given the high complexity and diversity of field environments, FPN assists the model in effectively handling such intricacies. Although MobileNetV1 is a lightweight model designed for resource-constrained devices, it may encounter performance bottlenecks when dealing with complex tasks. The utilization of FPN helps enhance the performance of MobileNetV1, enabling it to handle more intricate visual tasks. This helps the model better recognize the maturity of tobacco leaves in complex backgrounds. The fusion of multi-scale features assists the model in adapting to tobacco leaves of different sizes and shapes, thereby improving the model’s generalization ability and robustness under complex backgrounds. By combining MobileNetV1 with FPN, the multi-scale features of in situ tobacco leaves in the field are fully considered, which can improve the accuracy of tobacco leaf maturity classification while maintaining relatively low computational complexity.

#### 2.4.3. Integration of Spatial Attention Mechanism and SE Attention Mechanism

The integration of Spatial Attention (SP) [33] and Squeeze-and-Excitation (SE) [34] attention mechanisms into our model enhances its performance in recognizing field tobacco leaf maturity under complex weather and background conditions. SP guides the model to focus on more informative spatial regions in the input feature map, while the SE attention mechanism is a method of adaptively calibrating channel features in a convolutional neural network. It captures dependencies between feature channels, allowing the model to adaptively recalibrate channel feature responses, thereby enhancing feature expressiveness and classification accuracy.

**Figure 4:**
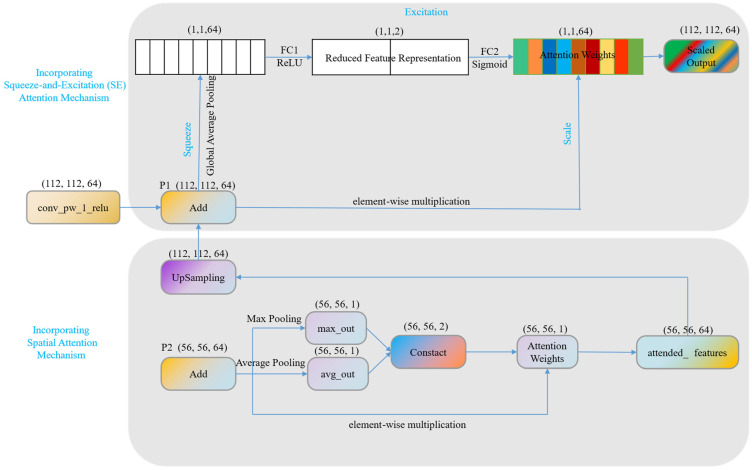
Integration of Spatial Attention mechanism and SE attention mechanism.

As shown in Figure 4, the SP mechanism was applied to the P2 layer of the FPN structure in our proposed model. By introducing SP, the model can automatically learn and pay attention to the most representative or informative parts of the input feature map, ultimately enhancing its expressiveness and performance under complex backgrounds. SP first compresses the input feature map through global average pooling and max pooling, generating two one-dimensional feature vectors. These two vectors represent the average and maximum response at each spatial location of the input feature map, generating an attention map. Then, these two feature vectors are concatenated to form a two-dimensional feature map, which is passed through a 3 × 3 convolution layer with a sigmoid activation function to generate a new weight map. Finally, the weight map is elementwise multiplied with the original input feature map to produce the final output feature map. This implements an adaptive weight adjustment for each spatial location of the input feature map, guiding the model to focus on important spatial regions.

The SE attention mechanism was applied to the P1 layer of the FPN structure. The workflow of the SE attention mechanism mainly includes three parts: Squeeze, Excitation, and Scale. In the Squeeze phase, a channel descriptor is generated using global average pooling, compressing each two-dimensional feature channel (H × W) into a real number, generating a one-dimensional vector that describes the global features of each channel. In the Excitation phase, the squeezed vector is nonlinearly transformed through a fully connected layer with ReLU activation and a fully connected layer with sigmoid activation, generating a weight vector reflecting channel dependencies. Finally, in the Scale phase, the weights are multiplied channel-wise with the original input feature map, implementing adaptive channel weight adjustment for the feature map. In this way, the SE attention mechanism can strengthen important channel features and suppress relatively unimportant channel features, further enhancing the performance of the model.

By integrating SP and SE attention mechanisms, the model benefits from the complementary advantages of spatial and channel recalibration. This combination allows the model to better adapt to different sizes and shapes of tobacco leaves and improves its generalization ability and robustness in complex background images. The resulting model is more effective in recognizing field tobacco leaf maturity under different weather conditions and background changes, helping to improve the performance of tobacco leaf maturity classification.

#### 2.4.4. Overview of the Enhanced MobileNetV1 Model

**Figure 5:**
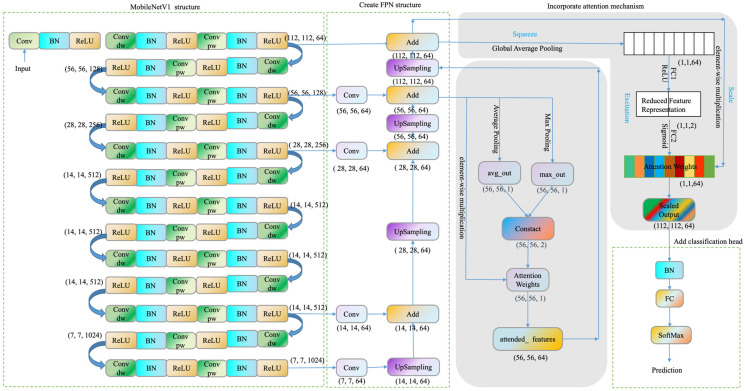
MobileNetV1 structure integrated with Feature Pyramid Network (FPN) and Spatial Attention and SE attention mechanisms.

We have made a series of improvements to the MobileNetV1 model, as shown in Figure 5, to enhance its performance in recognizing the maturity of tobacco leaves under complex backgrounds. Initially, feature maps C1, C2, C3, C4, and C5 were extracted from different layers of MobileNetV1. Then, by combining these feature maps with the FPN structure, we generated feature pyramids P5, P4, P3, P2, and P1 to achieve multi-scale feature fusion.

Further, the SP mechanism was applied in the P2 layer of the FPN structure, allowing the model to automatically focus on the more representative and informative parts of the input feature map. Then, the SE attention mechanism was applied in the P1 layer of the FPN structure to capture the inter-channel dependencies and adaptively recalibrate the channel-wise feature responses.

Finally, we added a BatchNormalization layer and a GlobalAveragePooling2D layer to the model to reduce spatial dimensions, followed by adding a fully connected layer with softmax activation to output predictions for nine categories of tobacco leaf maturity. By connecting the input of MobileNet with the predictive output, we created an enhanced MobileNetV1 model with FPN structure, and SP and SE attention mechanisms.

This enhanced model improves the accuracy of tobacco leaf maturity classification while maintaining low computational complexity. By introducing multi-scale feature fusion and attention mechanisms, the model can better adapt to different sizes and shapes of tobacco leaves, thereby improving its generalizability and robustness in complex background images. This approach takes full account of the multi-scale features of field tobacco leaves and helps to improve the performance of tobacco leaf maturity classification.

### 2.5. Model Training and Validation

#### 2.5.1. Training Parameters and Optimization Strategy

In this study, cross-entropy loss was employed as the loss function for training the model. The batch size was set to 32, and training was performed for 30 epochs. Furthermore, the learning rate was dynamically adjusted using the ReduceLROnPlateau strategy. If the loss on the validation set did not decrease after five consecutive epochs, the learning rate was halved.

#### 2.5.2. Training Parameters and Optimization Strategy

For comprehensive performance evaluation of the proposed in situ tobacco leaf maturity recognition model, several widely recognized and used metrics were adopted as evaluation indices, including accuracy, recall, precision, F1 score, mean average precision (*mAP*), frame rate (*FPS*), the number of model parameters, and weight size. Their formulas are as follows:

(1) $$\mathrm{Accuracy}=\frac{TP+TN}{TP+TN+FP+FN}$$

(2) $$Recall=\frac{TP}{TP+FN}$$

(3) $$Precision=\frac{TP}{TP+FP}$$

(4) $$F1=\frac{2\times (Precision\times Recall)}{Precision+Recall}$$

(5) $$AP=\int_{0}^{1}\;Precision(Recall)dRecall$$

(6) $$mAP=\frac{1}{n}\sum_{i=1}^{n}\;AP$$

(7) $$f_{FPS}=\frac{N}{t}$$

In these formulas, TP represents the number of true positive samples correctly predicted by the model, TN represents the number of true negative samples correctly predicted by the model, FP represents the number of false positive samples incorrectly predicted by the model, and FN represents the number of false negative samples incorrectly predicted by the model. *n* represents the number of image recognition categories, *N* represents the number of images recognized, and *t* represents the recognition time.

The accuracy, recall, precision, F1 score, and *mAP* mainly reflect the predictive accuracy and performance of the model. FPS indicates the computational efficiency of the model, which is crucial in real-time applications. The number of model parameters and weight size represent the complexity of the model and memory usage. These evaluation metrics provide a comprehensive understanding of the model performance in different aspects. By analyzing these indicators, we can better assess the suitability of the model for the tobacco leaf maturity recognition task in different scenarios and make further improvement decisions.

## 3. Results and Discussion

### 3.1. Experiment Environment and Parameters

The experiments were conducted in a Python 3.7 environment with TensorFlow-GPU version 2.6.0. Training was performed using the Pycharm compiler on a hardware environment featuring an 8-core i5-12450H processor and 16 GB of VRAM. The batch size was set to 32, and training was performed for 30 epochs.

### 3.2. Optimizer and Learning Rate Selection

Choosing the appropriate optimizer and learning rate has a significant impact on model performance and accuracy. In deep learning, the optimizer is responsible for controlling the update of model parameters, while the learning rate determines the magnitude of each parameter update. A learning rate that is too small can cause the objective function to decrease slowly, leading to slow model convergence or inability to converge to the optimal solution; a learning rate that is too large may cause oscillation near the optimal solution or lead to the explosion of the objective function, resulting in the model failing to converge or overfitting. The choice of optimizer also affects model performance and accuracy, as different optimizers have different characteristics and strengths.

To further investigate the impact of model parameters on network accuracy, we chose three optimizers—Stochastic Gradient Descent (SGD), RMSprop, and Adaptive Moment Estimation (Adam)—and applied them to the pre-trained MobileNetV1 tobacco leaf maturity recognition model integrated with an FPN and attention mechanism. The initial learning rates of 0.005, 0.0005, and 0.00005 were used for in situ tobacco leaf maturity recognition training. These three optimizers are the most commonly used and popular optimizers in the field of deep learning. They have achieved good performance in various types of neural networks. The Adam and RMSprop optimizers can adaptively adjust the learning rate, effectively controlling the size of parameter updates, and have faster convergence speeds and better generalization performance. In contrast, the SGD optimizer might require more training steps to reach the optimal solution but can avoid overfitting to some extent.

**Figure 6:**
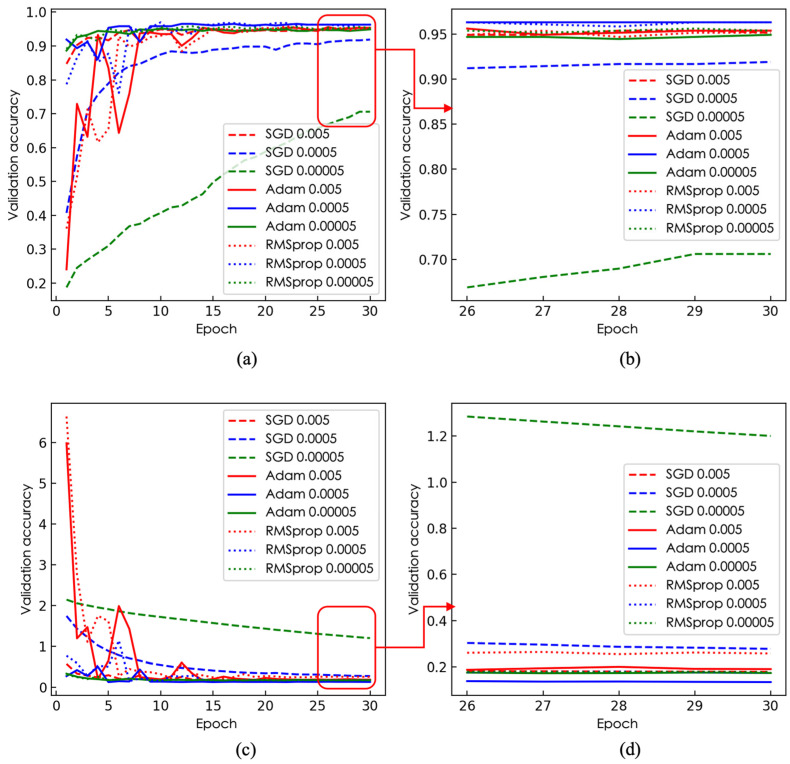
Comparison of accuracy and loss under different optimizers and learning rates: (**a**) accuracy under different optimizers and learning rates, (**b**) accuracy in the final five epochs under different optimizers and learning rates, (**c**) loss under different optimizers and learning rates, (**d**) loss in the final five epochs under different optimizers and learning rates.

The impact of the optimizer and learning rate on the improved MobileNetV1 network model is shown in Figure 6. The Adam optimizer outperformed the SGD and RMSprop optimizers at all learning rates. Especially at a learning rate of 0.0005, when the last five epochs were inspected, it can be seen that the Adam optimizer converged quickly and achieved the highest accuracy and lowest loss values, 96.3% and 0.13, respectively, indicating that the Adam optimizer achieved a good balance of learning speed and model performance. In comparison, although the SGD optimizer showed some accuracy at a learning rate of 0.005, its loss value was higher than that of Adam and RMSprop, and its convergence speed was slower. The RMSprop optimizer, at a learning rate of 0.0005, had accuracy and loss values similar to Adam but a moderate convergence speed. Overall, the Adam optimizer maintained the best performance and convergence speed at a learning rate of 0.0005. Therefore, when training the tobacco leaf maturity recognition model, we used the Adam optimizer and a learning rate of 0.0005 to achieve high performance in the shortest time.

### 3.3. Ablation Study

The aim of this study was to improve the accuracy of the MobileNetV1 model by introducing an attention mechanism and FPN layers. To verify the individual contributions of these improvements to the model and their impact on model performance, we conducted an ablation study, the results of which are presented in Table 1.

From Table 1, we can see that the MobileNetV1 model with the FPN structure had improved accuracy on both the validation and test sets, increasing from 95.37% to 96.06% and from 94.47% to 95.39%, respectively. This indicates that the FPN structure can effectively implement multi-scale feature fusion, enhancing the model’s generalization capability. When we introduced the SE attention mechanism on this basis, although the accuracy of the validation set decreased compared to the base model, the accuracy of the test set improved. This suggests that while the SE mechanism may lead to overfitting of the training data, it does indeed enhance the model’s recognition accuracy in complex scenarios by improving the model’s ability to adaptively calibrate channel features.

We can also see that when the SP mechanism was introduced, the model’s accuracy on the validation set further improved, but the accuracy on the test set decreased slightly. This may be due to the SP mechanism’s overfitting of the training data. However, when we integrated both the SP and SE attention mechanisms into the model, the accuracy on both the validation and test sets reached the highest values of 96.3% and 96.31%, respectively. This suggests that while there may be some issues when using SP and SE attention mechanisms independently, when they work together, they can complement each other, collectively enhancing model performance and achieving higher tobacco leaf maturity recognition performance, exhibiting an excellent generalization capability.

**Figure 7:**
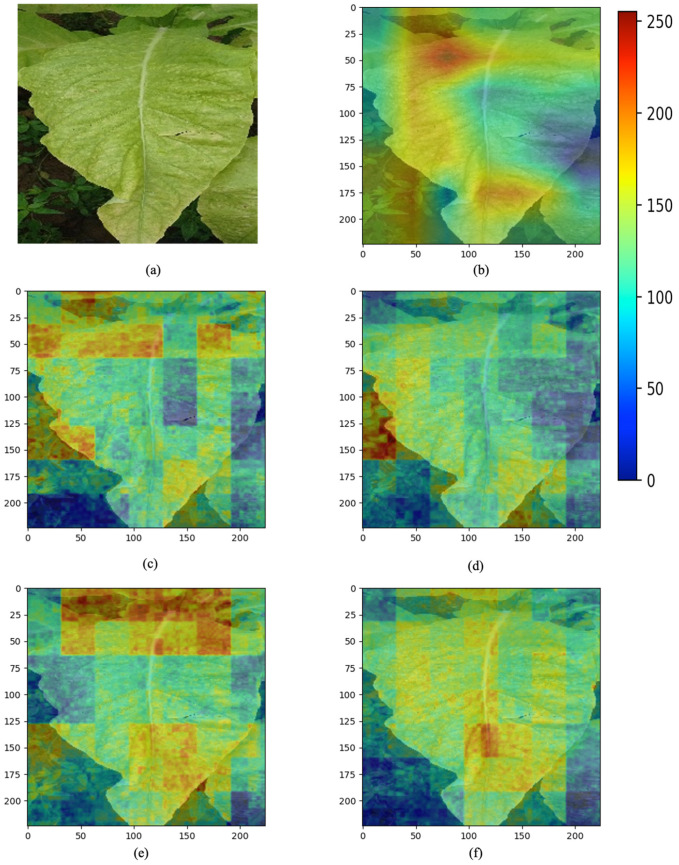
Overlay visualization of feature maps from different network structures and the original input image: (**a**) original image, (**b**) MobileNetV1, (**c**) MobileNetV1 + FPN, (**d**) MobileNetV1 + FPN + SE, (**e**) MobileNetV1 + FPN + SP, (**f**) MobileNetV1 + FPN + SP + SE.

To more intuitively show how these improvements affect the model’s decision-making process, we performed feature visualization analysis on the layer prior to the global average pooling layer of the model. This layer, which is close to the network output, contains more discriminative features. These features are crucial for the model’s classification decision and are located before the global average pooling operation, so the feature maps still have spatial dimensions. Therefore, we can see the distribution of each feature in the image. To understand how this layer responds to input images, we overlayed its output with the original input images. We employed a process of extracting the maximum activation value across all channels at each position to generate a new two-dimensional image, which was resized to match the original image. Subsequently, the grayscale values of the two-dimensional image were transformed into colors, and the original input image was blended with the color-mapped feature map. In the resulting image, as shown in Figure 7, blue areas indicate weaker responses from the model at these locations, while red areas indicate stronger responses. This blended display method can effectively reveal which areas of the image the model reacted strongly to, thereby helping us understand the model’s decision-making process.

Observations revealed significant differences in the feature visualization between the MobileNetV1 model with integrated FPN and the original model, particularly in the layer preceding the global average pooling. This disparity can be attributed to FPN serving as an efficient multiscale spatial feature extraction network capable of capturing image features across multiple scales. This characteristic enables FPN to extract more refined and diverse features from the image, such as clear edge and shape information. Consequently, the integrated FPN in the MobileNetV1 model may present a greater abundance of line information, resembling a heatmap created by line contours.

Subsequently, attempts were made to further incorporate SP mechanisms and SE attention mechanisms into the FPN-based model. However, these improvements, when applied to the recognition of tobacco leaf maturity, exhibited varying degrees of excessive focus on background elements, such as weeds. In contrast, the enhanced model that integrates FPN, SP mechanisms, and SE attention mechanisms primarily emphasized the features of the tobacco leaves themselves, providing a more favorable basis for tobacco leaf maturity recognition.

These experimental results highlight the significance of FPN, SP, and SE mechanisms in the improved model. FPNs, by integrating features at different scales, help the model capture richer contextual information. The SE enhances the model’s recognition accuracy in complex scenarios by modeling dependencies between channels. The SP enables the model to automatically focus on important parts of the input feature map. When these improvements are combined, the model demonstrates significant performance improvements in identifying the maturity of tobacco leaves under complex weather and background conditions.

### 3.4. Performance Evaluation of Different Models

To further verify the recognition performance of the improved model, we conducted recognition tests on the maturity of tobacco leaves and compared it with classic models such as MobileNetV1, MobileNetV2, MobileNetV3, VGG16, VGG19, ResNet50, EfficientNetB0, and EfficientNetB1. The comparison results are shown in Table 2.

Firstly, in terms of recognition accuracy, both MobileNetV3Large and the enhanced version of MobileNetV1 stood out, achieving a remarkable test accuracy of up to 96.3%, positioning them as leaders in the field. They were closely followed by EfficientNetB1 and MobileNetV3Small, achieving 95.85% and 94.93%, respectively. In precision and recall, the enhanced MobileNetV1 model reaffirmed its exceptional performance, with respective scores of 96.47% and 96.31%. This underscores the model’s robust capacity to correctly identify true positives and retrieve the majority of actual positive cases. The MobileNetV3Large model ranked second, with precision and recall rates of 96.31% and 96.29%, respectively. The F1 score, a balanced measure of precision and recall, was led by the enhanced MobileNetV1, achieving 96.33%, slightly below MobileNetV3Large’s score of 96.46%. Subsequent models include EfficientNetB1 and MobileNetV1, with F1 scores of 95.79% and 94.46%, respectively. Regarding mean average precision, the top performers were the enhanced MobileNetV1 and MobileNetV3Large, both scoring 96.31%, followed by MobileNetV1 and MobileNetV3Small, at 94.47% and 94.93%, respectively. This metric highlights the model’s aptitude for accurate pixel classification within the image.

Secondly, regarding model weight size, MobileNetV3Small stood out as the smallest model, at only 6.7 M. In terms of model size, MobileNetV2 closely follows with a size of 9.5 M, succeeded by MobileNetV1 and its enhanced version, sized at 13.2 M and 13.7 M, respectively. These compact models have distinct advantages in storage-constrained environments. Lastly, in terms of processing speed (FPS), MobileNetV1 achieved the highest speed, with MobileNetV2 and the enhanced MobileNetV1 following closely behind.

In conclusion, the enhanced MobileNetV1 model exhibits superior performance across several metrics, including test accuracy, precision, recall, F1 score, and mean average precision. While the enhanced MobileNetV1 and MobileNetV3Large attained identical recognition accuracy, the former lags slightly in terms of processing speed and model weight size compared to MobileNetV1 and MobileNetV2. However, it still significantly outperforms traditional models such as MobileNetV3Large, ResNet50, and VGG16. This demonstrates that the enhanced MobileNetV1, despite maintaining high performance, necessitates less storage and computational complexity. This suggests that this enhanced model holds significant advantages and broad application potential in this task. All in all, the enhanced MobileNetV1 proved to be an effective and efficient model, ideally suited for recognizing tobacco leaf maturity in complex in situ field conditions and backgrounds, especially in resource-constrained environments.

### 3.5. Model Robustness Evaluation against Real-World Application Challenges

Model robustness is an important metric to assess its sensitivity to small variations in input data. Research on model robustness primarily focuses on how to reduce the model’s sensitivity to input data without compromising model performance. In the practical application of on-site tobacco leaf maturity recognition, the complex field environment imposes high demands on the model’s generalizability. Common environmental disturbances include noise such as raindrops or mud spots on tobacco leaves, which add uncertainty and randomness to the environment. In addition, changes in ambient light can cause changes in image brightness, which is an important factor affecting image recognition. Furthermore, in practical applications, the target object may be partially or completely obscured by other objects. In our research, tobacco leaves might overlap, causing occlusion.

Therefore, we comprehensively evaluated and improved the model’s robustness through three types of experiments (noise disturbance, brightness transformation, and occlusion) to cope with the complex and changing environmental conditions in practical applications. In the noise disturbance experiment, Gaussian noise was selected with noise intensities of 0.5 and 0.1. In the brightness transformation experiment, brightness intensities of 0.8 and 1.2 were used. In the occlusion experiment, occlusion ratios of 0.3 and 0.5 were adopted. For each experiment, we conducted experiments with dataset proportions of 30%, 70%, and 100% to ensure the comprehensiveness of the results. These experiments aimed to simulate various challenges that might be encountered in real-world situations, hoping that the model could still maintain high performance when facing these challenges.

**Figure 8:**
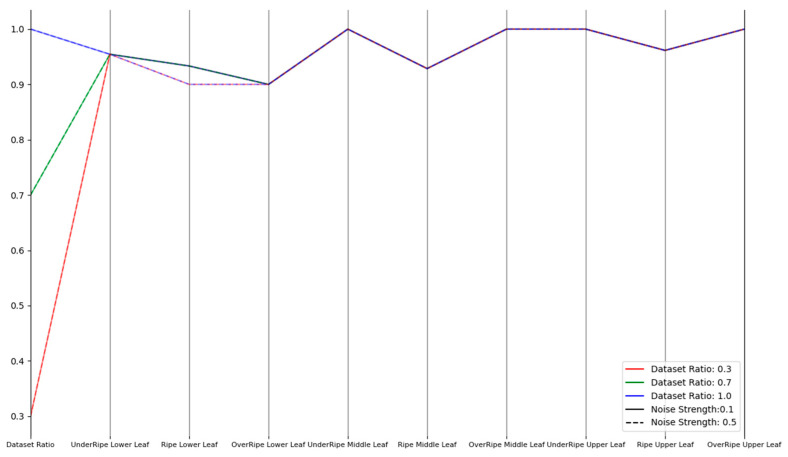
Parallel coordinates plot for noise disturbance experiment.

**Figure 9:**
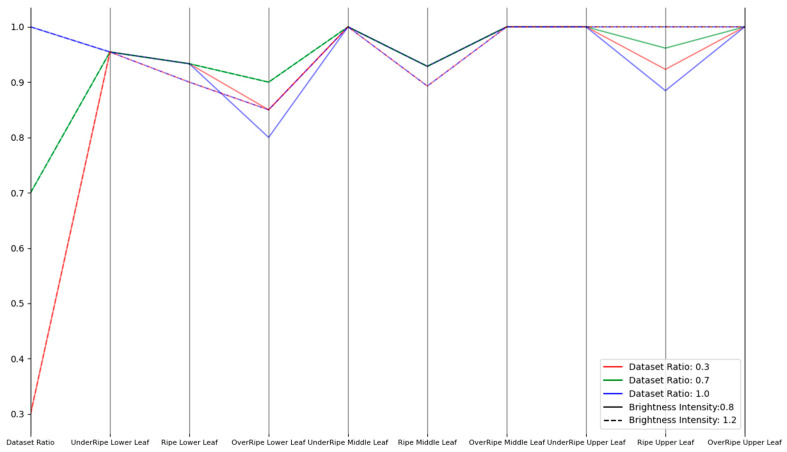
Parallel coordinates plot for brightness transformation experiment.

**Figure 10:**
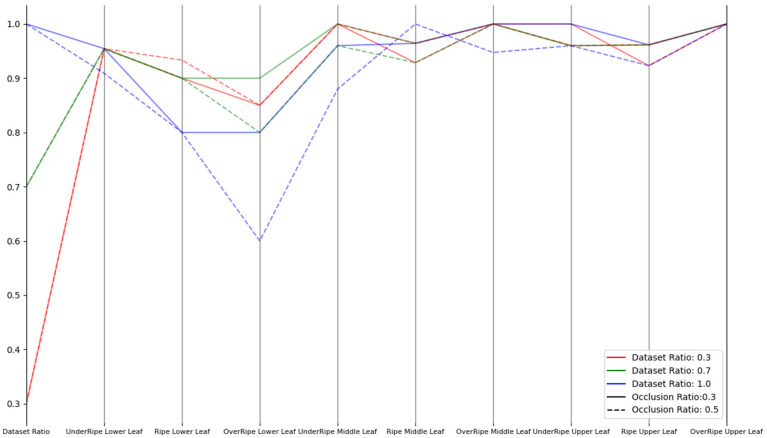
Parallel coordinates plot for occlusion experiment.

As shown in Figure 8, Figure 9 and Figure 10, the improved model demonstrated good robustness under different conditions of noise intensity, brightness changes, and occlusion ratios, from the experimental data. Whether for lower, middle, or upper leaves, at various maturity stages, the model’s recognition accuracy was maintained at a high level. For the noise disturbance experiment, even when the noise intensity increased to 0.5 and the noise accounted for more than 70%, the model could still maintain a relatively high accuracy, with only a slight decrease in the recognition of mature leaves. In the brightness transformation experiment, the model could stably identify the maturity of tobacco leaves, whether the brightness intensity was 1.2 or 0.8. At a brightness intensity of 0.8, the recognition accuracy for mature upper leaves and overripe lower leaves slightly decreased, possibly due to the loss of information caused by low brightness. In the occlusion robustness test, the model performed well. Even under extensive occlusion, the recognition accuracy in most cases remained high. However, the recognition of mature and overripe lower tobacco leaves declined when the occlusion ratio was 0.5, and the proportion of occluded dataset was 100%, likely due to the loss of important information for maturity judgment caused by excessive occlusion. Overall, the improved model demonstrated good robustness against noise, occlusion, and brightness changes, and could accurately identify tobacco leaves of different positions and maturities.

### 3.6. Analysis and Visualization of Maturity Recognition Results

To analyze the performance of the improved model in recognizing different maturity levels of tobacco leaves in different parts of the plant, we used a confusion matrix and Score-CAM visualization methods to display and interpret the model’s prediction results.

**Figure 11:**
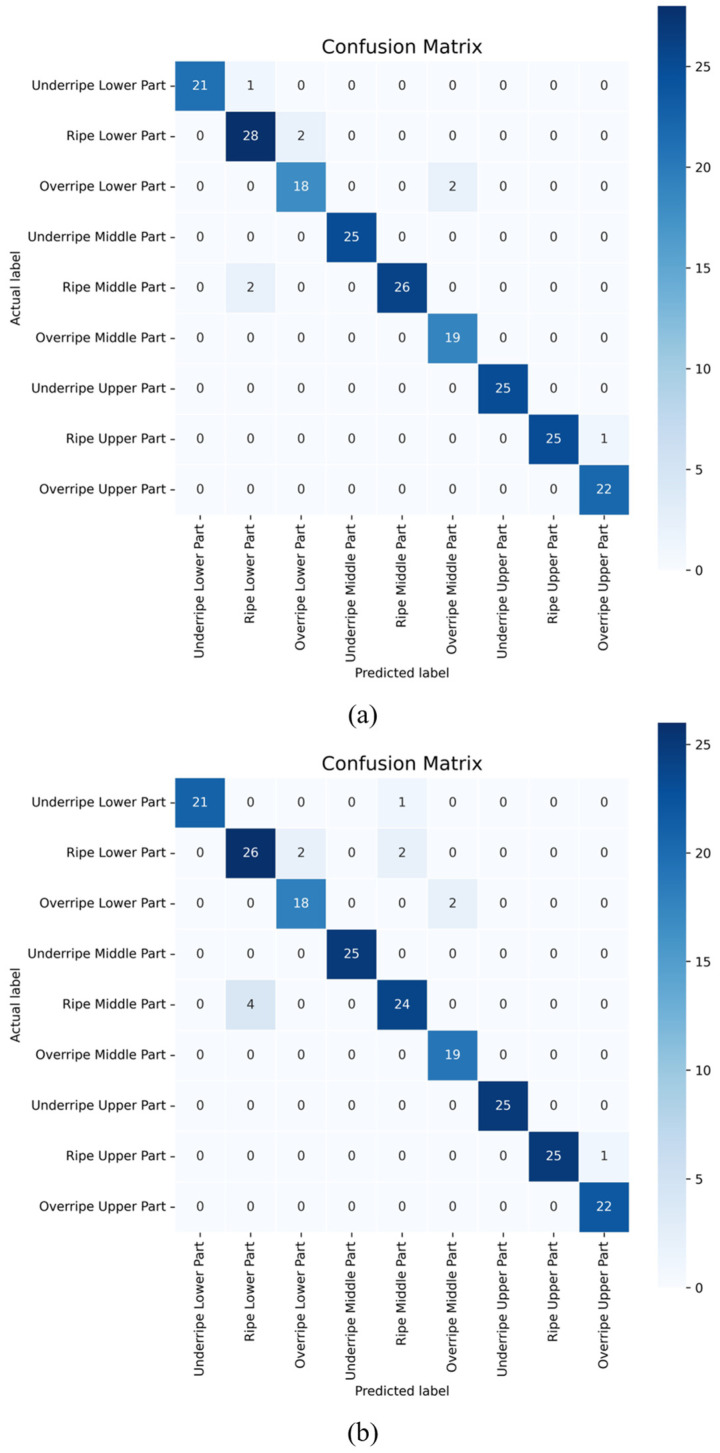
Confusion matrices of the original and improved MobileNetV1 models: (**a**) improved model, (**b**) original model.

From the confusion matrices of the model before and after improvements in Figure 11, we can observe that the improved model performed well in all categories, especially in recognizing the categories of lower immature, middle immature, middle overripe, upper immature, and upper overripe leaves. Only a few samples made errors in classification, and only a few samples were misclassified when identifying the categories of lower mature, middle mature, and upper mature. In the same task, although the original model also performed well in recognizing the categories of lower immature, middle immature, middle overripe, upper immature, and upper overripe, there were many misclassified samples when dealing with complex categories of lower mature and middle mature. In general, the improved model performed better and made fewer misclassifications when dealing with the two more complex categories of lower mature and middle mature. This highlights the effectiveness and applicability of the improved model, especially when handling complex category recognition tasks.

The Score-CAM (Score-weighted Class Activation Mapping) [35] algorithm can provide profound insights into the contributions of deep learning models in recognizing the maturity of fresh tobacco. Score-CAM, a visualization technique, can predict behaviors in Convolutional Neural Networks (CNNs), and is particularly apt for Softmax classification models. In comparison to other methods, Score-CAM demonstrates reduced computational complexity, crucial for managing complex backgrounds and resource-constrained devices. Additionally, Score-CAM does not necessitate gradient information, thereby circumventing potential noise and instability issues that may surface with Grad-CAM. By generating Class Activation Maps (CAMs), it aids in explicating the model’s key feature location, localization accuracy, performance, and interpretability in the process of recognizing the maturity of fresh tobacco, thereby assessing whether the model captures salient features pertaining to specific categories.

The fundamental principle of Score-CAM is to weigh activation images based on the scores of the target category, thereby generating an interpretable heatmap. Initially, during the forward propagation process, the model generates a series of activation images for the input image at a particular convolutional layer. Subsequently, each activation image is propagated forward up to the Softmax layer to compute the corresponding category score. These calculated category scores are then used to weigh these activation images to derive the final class activation mapping. Throughout this procedure, Score-CAM can yield highly discriminative and precise localization results.

**Figure 12:**
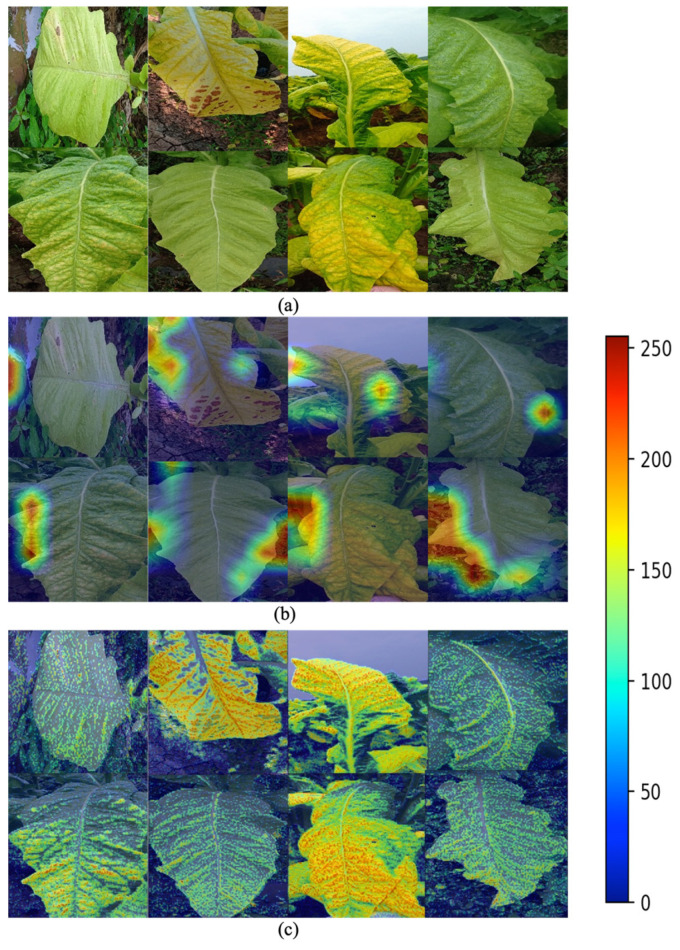
Comparative visualization of Score-CAM recognition results: (**a**) original image, (**b**) visualization of the Score-CAM recognition results using the original model, (**c**) visualization of the Score-CAM recognition results using the improved model.

The pixels within the heatmap correspond to regions on the input image, indicative of the model’s focus on that area. Typically, heatmaps utilize color-coding to signify weights or intensities, where warmer colors (such as red) imply greater attention paid by the model during prediction, and cooler colors (like blue) indicate less attention. The results of the fresh tobacco maturity recognition task of the pre- and post-improved model are visualized using Score-CAM. As illustrated in Figure 12, when the MobileNetV1 model was amalgamated with the Feature Pyramid Network (FPN) and attention mechanisms, the model’s focal point shifted, and the improved model exhibited enhanced precision in localization. Compared to the original MobileNetV1 model, the highlighted regions in the heatmap focused more on the color and texture of the tobacco leaves relevant to maturity judgement, especially the areas of tobacco leaf veins and color changes. This is particularly evident in mature and overripe tobacco leaves, which assists in enhancing classification accuracy.

### 3.7. Validation of the Enhanced MobileNetV1 on the V2 Plant Seedlings Dataset

To validate the performance of our enhanced model, we applied it to the widely available V2 Plant Seedlings Dataset. This dataset, composed of 5539 RGB images captured under a variety of weather conditions, closely mirrors the real-world scenarios encountered by our research subject: in-field tobacco leaves. It encompasses three species of plants and nine types of weeds.

The dataset was divided into training, validation, and testing subsets at ratios of 70%, 15%, and 15%, respectively. The training subset comprised 3877 images, including three plant species—common wheat, maize, and sugar beet—and nine weed species, namely, black-grass, common chickweed, cleavers, scentless mayweed, small-flowered cranesbill, shepherd’s purse, loose silky-bent, charlock (also known as wild mustard), and fat hen. The validation and testing subsets, each containing approximately 831 images, similarly included the same range of plant and weed species.

Through comparative analysis, as illustrated in Table 3, our enhanced model demonstrated an accuracy of 96.63% in the plant seedling recognition task, outperforming other models. This result validates the model’s effectiveness and superiority.

## 4. Discussion

In tackling the challenge of tobacco leaf maturity recognition within complex field environments, our study proposed a model that incorporates MobileNetV1, FPN, and attention mechanisms. This model exhibited outstanding performance in maturity recognition tasks, achieving an accuracy rate of 96.3%, and demonstrating significant robustness. However, as deep learning technologies continue to advance, a host of novel network structures and techniques have emerged. Innovative network architectures such as Mobile-Former [39], MixFormer [40], TopFormer [41], EfficientFormer [42], RepVGG [43], and LeVit [44] may offer considerable enhancements for such tasks.

Mobile-Former and MixFormer utilize feed-forward transformer structures, promising potential advancements in computational efficiency and model accuracy. Conversely, RepVGG and LeVit offer more streamlined and efficient convolutional neural networks and visual transformer structures, potentially further optimizing model performance. These novel network architectures could provide superior performance and adaptability, warranting further investigation in future research.

## 5. Conclusions

In addressing the challenge of distinguishing tobacco leaf maturity in complex field environments, we have developed a tobacco leaf maturity classification model. This model leverages an enhanced MobileNetV1 framework, a FPN, and an attention mechanism. It demonstrated resilience and precision in tackling tobacco leaf maturity identification challenges within intricate field settings, achieving an accuracy rate of 96.3%, which surpassed conventional models such as VGG16, VGG19, ResNet50, and EfficientNetB0. Furthermore, it attained a 96.63% accuracy rate on the V2 Plant Seedlings Dataset. Vein patterns and color transition zones in tobacco leaves emerged as critical features in maturity recognition.

Our refined MobileNetV1 model, while delivering superior performance, requires minimal storage and computational power, signifying its substantial potential for real-world application in tobacco leaf maturity recognition within the field. Future research could delve deeper into updated backbone network architectures like Mobile-Former, MixFormer, TopFormer, EfficientFormer, RepVGG, and LeVit, which could potentially enhance model performance and expedite the advancement of tobacco leaf maturity recognition technology.

In conclusion, our investigation presents a practical solution for the automatic discernment of tobacco leaf maturity, making a significant contribution to the burgeoning field of smart agriculture.

## Figures and Tables

**Table 1:** Accuracy on validation and test sets for different network architectures.

Model	Validation Set Accuracy/%	Test Set Accuracy/%
MobileNetV1	95.37	94.47
MobileNetV1 + FPN	96.06	95.39
MobileNetV1 + FPN + SE	95.14	94.93
MobileNetV1 + FPN + SP	96.3	94.01
MobileNetV1 + FPN + SP + SE	96.3	96.31

**Table 2:** Comparative analysis of different models.

Model	Test Set Recognition Accuracy	Precision	Recall	F1	Number of Parameters	Model Weight Size (M)	FPS	mPA
VGG16	0.7972	0.8058	0.7972	0.7986	134,297,417	537.2	48.14	79.72
VGG19	0.7788	0.7849	0.7788	0.7781	139,607,113	558.5	39.82	77.88
ResNet50	0.9217	0.9206	0.9217	0.9203	23,606,153	94.9	77.19	92.17
EfficientNetB0	0.9355	0.937	0.9355	0.9359	4,061,100	16.8	74.58	59.91
EfficientNetB1	0.9585	0.9595	0.9585	0.9579	6,586,768	27.1	59.37	59.91
MobileNetV1	0.9447	0.9453	0.9447	0.9446	3,238,089	13.2	196.41	94.47
MobileNetV2	0.9401	0.9451	0.9402	0.9422	2,269,513	9.5	133.63	94.01
MobileNetV3Small	0.9493	0.9513	0.9542	0.9525	1,539,193	6.7	145.66	94.93
MobileNetV3Large	0.9631	0.9631	0.9629	0.9646	4,237,961	17.7	107.18	96.31
Improved MobileNetV1	0.9631	0.9647	0.9631	0.9633	3,357,276	13.7	128.12	96.31

**Table 3:** Comparison of a proposed method with the existing models.

Method	Model	Test Set Accuracy (%)
[36]	Faster R-CNN-FPN	95.61
[37]	ResNet50	96.21
[38]	EfficientNetB0	96.52
Improved MobileNetV1	MobileNetV1	96.63

## Data Availability

Not applicable.

## Abbreviations

CNN	Convolutional Neural Network
FPN	Feature Pyramid Network
SE	Squeeze and Excitation
SP	Spatial Attention
